# Coexistence of Tripartite Accessory Navicular Bone and Os Subfibulare

**DOI:** 10.3390/diagnostics16121838

**Published:** 2026-06-13

**Authors:** George Triantafyllou, Nikolaos-Achilleas Arkoudis, Christos Koutserimpas, Spyridon Prountzos, George Tsakotos, Maria Piagkou, Olympia Papakonstantinou

**Affiliations:** 1 Department of Anatomy, School of Medicine, Faculty of Health Sciences, National and Kapodistrian University of Athens, 11527 Athens, Greece; georgerose406@gmail.com (G.T.); gtsakotos@gmail.com (G.T.); mapian@med.uoa.gr (M.P.); 2 Research Unit of Radiology and Medical Imaging, National and Kapodistrian University of Athens, 11527 Athens, Greece; nick.arkoudis@gmail.com (N.-A.A.); sogofianol@gmail.com (O.P.); 3 Second Department of Radiology, General University Hospital “Attikon”, National and Kapodistrian University of Athens, 12462 Athens, Greece; spyttt@gmail.com; 4 School of Health Rehabilitation Sciences, University of Patras, 26504 Patras, Greece

**Keywords:** accessory navicular bone, os subfibulare, os trigonum, musculoskeletal radiology, variation

## Abstract

This report describes a unique constellation of accessory ossicles, highlighting their anatomical, clinical, and radiological significance. A 43-year-old female undergoing imaging for suspected fracture was evaluated using multi-detector computed tomography (MDCT) with 1.25 mm slice thickness. Multiplanar reconstructions (axial, coronal, sagittal) and three-dimensional volume-rendered images were analyzed. CT imaging revealed the coexistence of an os subfibulare and a tripartite os naviculare. Multiplanar and three-dimensional reconstructions confirmed the presence and configuration of variants. The combination of supernumerary bones and a multipartite ossicle represents an exceedingly uncommon anatomical presentation. This case illustrates an exceptional coexistence of multiple accessory ossicles, including an exceedingly rare tripartite os naviculare. Thorough radiological evaluation using MDCT and multiplanar reconstructions is essential for accurate identification and differentiation from fractures or other pathology.

**Figure 1:**
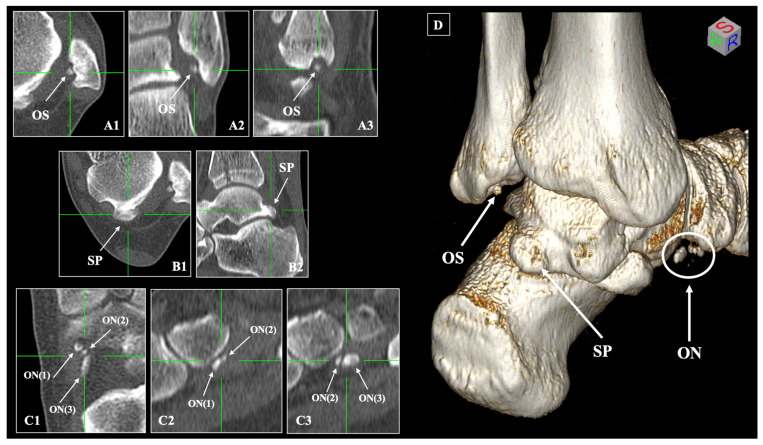
Axial (**A1**), coronal (**A2**) and sagittal (**A3**) reconstructions of the os subfibulare (OS). Axial (**B1**) and sagittal (**B2**) reconstructions of a Stieda’s process (SP). Axial (**C1**) and sagittal (**C2**,**C3**) reconstructions of the tripartite (1, 2, 3) os naviculare (ON). Three-dimensional reconstruction (**D**) demonstrating the coexistence of all variants. The scan belonged to a 43-year-old female patient who underwent MDCT scan with 1.25 slice thickness of the talus (after falling down the stairs) that showed a non-displaced fracture of the tibial plafond (Pilon fracture, type 1) (Supplementary Video S1). Axial (**A1**), coronal (**A2**) and sagittal (**A3**) reconstructions demonstrated the presence of an os subfibulare. This accessory ossicle measured 2.46 mm in diameter and was located 0.72 mm medial to the fibula. Axial (**B1**) and sagittal (**B2**) reconstructions demonstrated the presence of an elongated posterior lateral tubercle (Stieda’s process). It measured 8.48 mm in anteroposterior length and 6.96 mm in craniocaudal length. Axial (**C1**) and sagittal (**C2**,**C3**) reconstruction images demonstrated the presence of a tripartite os naviculare. The anterior ossicle measured 2.76 mm in diameter, the middle ossicle measured 1.68 mm, and the posterior ossicle measured 4 mm. All ossicles were well corticated. Lastly, three-dimensional reconstruction (**D**) confirmed the presence of these accessory ossicles. All accessory ossicles were rounded and well corticated. The rarest finding in the current case was the tripartite os naviculare. The accessory navicular bone has a pooled prevalence of 12.6% according to the latest meta-analysis, which represents a common finding [1]. However, multipartite variants are rarely reported in the literature. Sharkey et al. [2] reported that they identified the first case of bipartite os naviculare (two accessory navicular bones) in a symptomatic child who presented with sharp pain which required surgical excision of the ossicle. However, Perdikakis et al. [3] reported three cases of bipartite os naviculare and two cases of tripartite os naviculare in a sample of 170 ankles. Therefore, we report a rare case of tripartite os naviculare that was previously described by Perdikakis et al. (2011), without coexistence of additional accessory ossicles [3]. The accessory navicular, although common, becomes clinically significant when symptomatic because of its relationship with the tibialis posterior tendon and the potential to provoke medial foot pain, tendon dysfunction, or flatfoot deformity [1]. Multipartite forms, while they are exceedingly rare, and the tripartite os naviculare in this case reflect an exceptional developmental variant with an unclear but potentially heightened risk for synchondrosis irritation or altered tendon biomechanics [2,3]. Furthermore, the elongated posterior lateral tubercle (Stieda’s process) is a finding that needs careful definition. Some authors define it as fused os trigonum; however, this term should be avoided. When a bony connection with the talus is observed, it corresponds to a Stieda’s process [4]. The coexistence of ossicles is a rare entity. Large retrospective radiological studies reported the coexistence of two accessory ossicles in 3.57–4.4% [5,6]. Although each ossicle alone is relatively well-defined anatomically, their simultaneous presence may have cumulative effects on regional biomechanics, tendon vector forces, and joint kinematics [6]. In such rare anatomical combinations, even if individual ossicles appear asymptomatic, their combined anatomical presence may complicate clinical evaluation, obscure the diagnosis of acute injury, or increase the likelihood of misinterpreting normal variants as fractures. Accurate radiological assessment is essential for identifying accessory ossicles and distinguishing them from acute fractures, avulsion injuries, or degenerative changes. Standard radiographs provide an initial overview, but may fail to delineate small, multipartite, or anatomically complex ossicles, particularly in regions with overlapping anatomical structures [7]. Multi-detector CT with thin-slice acquisition and multiplanar reconstructions offers superior spatial resolution for delineating cortical margins, determining osseous continuity, and confirming multipartite morphology [8]. Three-dimensional reconstructions further enhance visualization of spatial relationships, which is critical when multiple accessory bones coexist. MRI plays a complementary role by evaluating bone marrow edema, synchondrosis integrity, and associated soft-tissue pathology [8].

## Data Availability

The data presented in this study are available on request from the corresponding author.

## Supplementary Materials

The following supporting information can be downloaded at: https://www.mdpi.com/article/10.3390/diagnostics16121838/s1.
